# Luminescent properties of CdTe quantum dots synthesized using 3-mercaptopropionic acid reduction of tellurium dioxide directly

**DOI:** 10.1186/1556-276X-8-253

**Published:** 2013-05-29

**Authors:** Mao Shen, Wenping Jia, Yujing You, Yan Hu, Fang Li, Shidong Tian, Jian Li, Yanxian Jin, Deman Han

**Affiliations:** 1 College of Pharmaceutical and Chemical Engineering, Taizhou University, Jiaojiang 318000, People's Republic of China; 2 Department of Dyeing and Chemistry, Chengdu Textile College, Chengdu 611731, People's Republic of China; 3 State Key Laboratory of Chemical Resources Engineering, Beijing University of Chemical Technology, Beijing 100029, People's Republic of China

**Keywords:** CdTe quantum dots, 3-mercaptopropionic acid, TeO_2_, Microwave irradiation, Photoluminescence

## Abstract

A facile one-step synthesis of CdTe quantum dots (QDs) in aqueous solution by atmospheric microwave reactor has been developed using 3-mercaptopropionic acid reduction of TeO_2_ directly. The obtained CdTe QDs were characterized by ultraviolet–visible spectroscopy, fluorescent spectroscopy, X-ray powder diffraction, multifunctional imaging electron spectrometer (XPS), and high-resolution transmission electron microscopy. Green- to red-emitting CdTe QDs with a maximum photoluminescence quantum yield of 56.68% were obtained.

## Background

In recent years, water-soluble CdTe luminescent quantum dots (QDs) have been used in various medical and biological imaging applications because their optical properties are considered to be superior to those of organic dyes [1-4]. Up to now, in most of the aqueous approaches, Te powder was used as the tellurium source and NaBH_4_ as the reductant, which needs a pretreatment to synthesize the unstable tellurium precursor. The process of preparing CdTe QDs requires N_2_ as the protective gas at the initial stage [5-10]. Even though Na_2_TeO_3_ as an alternative tellurium source can also be used for preparing CdTe QDs [11-15], it is toxic and expensive. Therefore, it is very necessary to hunt for a novel tellurium source for the synthesis of CdTe QDs. Compared with Na_2_TeO_3_, TeO_2_ has the same oxidation state of Te and is stable, cheap, and less toxic. Recently, TeO_2_ was explored as the Te source for synthesis of CdTe QDs, but the reduction of TeO_2_ by NaBH_4_ in ambient conditions requires a long reaction time and easily produces a black precipitate of CdTeO_3_[16-20]. Here, we proposed a new facile synthetic approach for preparing CdTe QDs with tellurium dioxide as a tellurium source. 3-mercaptopropionic acid was explored as both reductant for the reduction of TeO_2_ and capping ligand for CdTe QDs. Such synthetic approach eliminates the use of NaBH_4_ and allows facile one-pot synthesis of CdTe QDs.

## Methods

### Chemicals

Tellurium dioxide (TeO_2_, 99.99%), cadmium chloride hemi(pentahydrate) (CdCl_2_ · 2.5H_2_O, 99%), and 3-mercaptopropionic acid (MPA, 99%) were purchased from Aldrich Corporation (MO, USA). All chemicals were used without additional purification. All the solutions were prepared with water purified by a Milli-Q system (Millipore, Bedford, MA, USA).

### Synthesis of CdTe QDs

In our experiments, 2 mmol CdCl_2_ · 2.5H_2_O was dissolved in 100 mL of deionized water in a breaker, and 5.4 mmol MPA was added under stirring. The pH of the solution was then adjusted to 10.0 by dropwise addition of 1 mol/L NaOH solution. Under stirring, 0.5 mmol TeO_2_ was added to the original solution. The typical molar ratio of Cd^2+^/Te^2−^/MPA was 1:0.25:2.7. The monomer was heated in a XO-SM100 microwave-assisted heating system (XO-SM100 Microwave and Ultrasonic combination response system, MW-50%; Xianou Company, Nanjing, China) and refluxed at different times to control the size of the CdTe QDs. The particles were extracted by precipitation with the addition of 2-propanol to the solution. Then, the resulting powders were dried at room temperature.

### Characterization

The absorption and photoluminescence (PL) spectra were measured using a UV-2501PC spectrometer (Shimadzu Corporation, Tokyo, Japan) and CARY ECLIPSE (Agilent Technologies, Santa Clara, USA) fluorescence spectrometer, respectively. The PL quantum yield was determined using Rhodamine 6G as fluorescence standard. X-ray powder diffraction (XRD) analysis was performed using a Dmax-2500 (CuK*α* = 1.5406 Å; Rigaku Corporation, Tokyo). The morphology of the QDs was characterized using JEM-2100 transmission electron microscopy (HR-TEM; Jeol Ltd., Tokyo). X-ray photoelectron spectra (XPS) were recorded by Thermo ESCALAB 250XI X-ray photoelectron spectrometer (Thermo Fisher Scientific Inc., Waltham, MA, USA) with nonmonochromatized Al K*α* radiation as excitation source.

## Results and discussion

**Figure 1:**
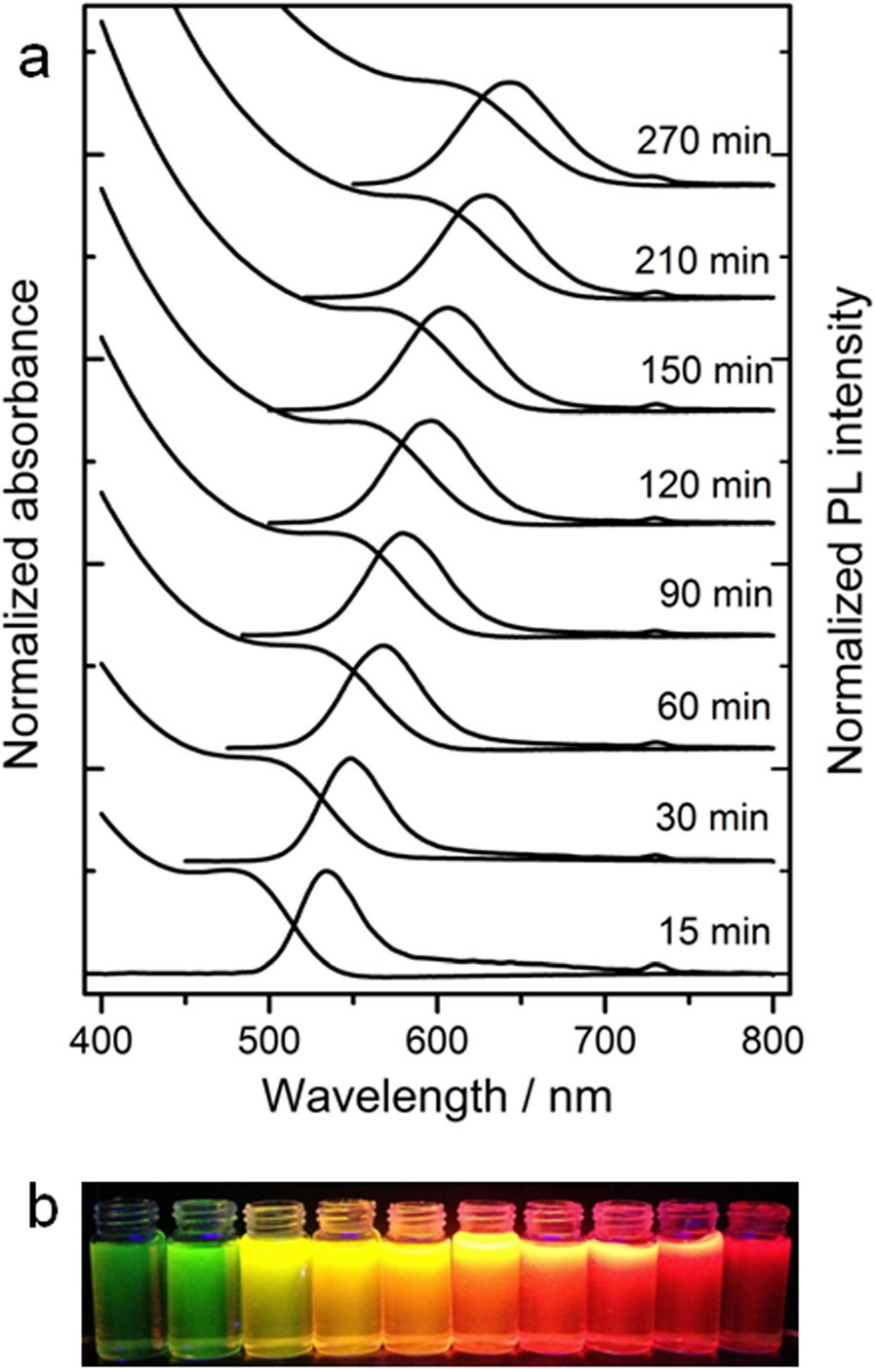
**Absorption, PL, and fluorescence emission spectra.** (**a**) Absorption and PL spectra (*λ*_ex_ = 365 nm) of CdTe QDs with different reflux times; (**b**) fluorescence emission spectra of CdTe QDs under UV (365 nm) irradiation. The maximum emission wavelengths of the CdTe QDs in (**a**) were 525 nm (15 min), 544 nm (30 min), 559 nm (60 min), 579 nm (90 min), 588 nm (120 min), 599 nm (150 min), 625 nm (210 min), and 641 nm (270 min), respectively.

The typical absorption PL spectra of CdTe QDs obtained with different refluxing times were given in Figure 1a. The redshifts of the absorption edge and the maximum PL emission wavelength indicated the growth of CdTe QDs during the heating treatment. The sizes of the QDs could be estimated from the UV–vis absorption spectrum by Yu and colleagues' empirical equation [21]:

$$\begin{array}{l} D=\left(9.8127\times 10^{-7}\right)\lambda^{3}-\left(1.7147\times 10^{-3}\right)\lambda^{2}\\ \;+\left(1.0064\right)\lambda -\left(194.84\right), \end{array}$$

where *λ* is the first absorption maximum. The diameters of the QDs ranged from 2.27 to 3.44 nm, indicating that the size of the QDs could be facilely tuned by varying the heating time. The fluorescent color under UV irradiation changed from green to yellow, orange, and finally to red with increasing heating time (Figure 1b).

**Figure 2:**
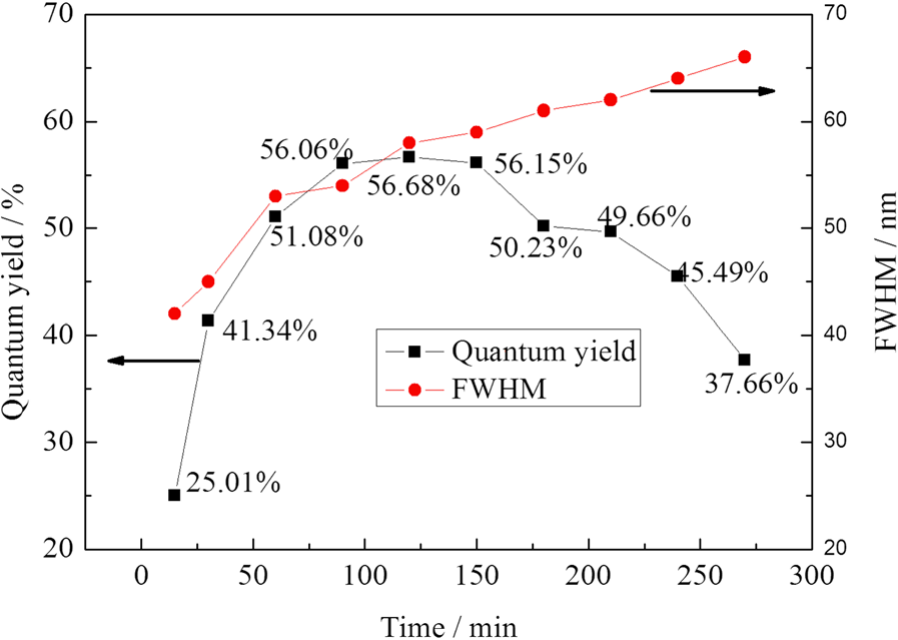
Variation of quantum yield and FWHM of CdTe QDs at different reflux times.

The PL quantum yield also depended on heating time (Figure 2). Increasing the heating time led to increased PL quantum yield, and maxima occurred at 120 min. Such PL quantum yield increase could be ascribed to the improvement of the crystallization and annealing effect of defects. However, further heating resulted in a decrease in PL quantum yield due to broad distribution and relatively small surface/volume ratio of the obtained QDs. Another evidence of the broad distribution is the increased full width at half maximum (FWHM) of the resultant CdTe QDs, which broadened from 40 to 66 nm in the heating time of 0 to 270 min. With heating time longer than 300 min, there were lots of black depositions in the solution, which may be caused by the oxidization and aggregation of CdTe QDs due to the destruction of MPA. Meanwhile, the PL quantum yield of the CdTe QDs decreases dramatically.

**Figure 3:**
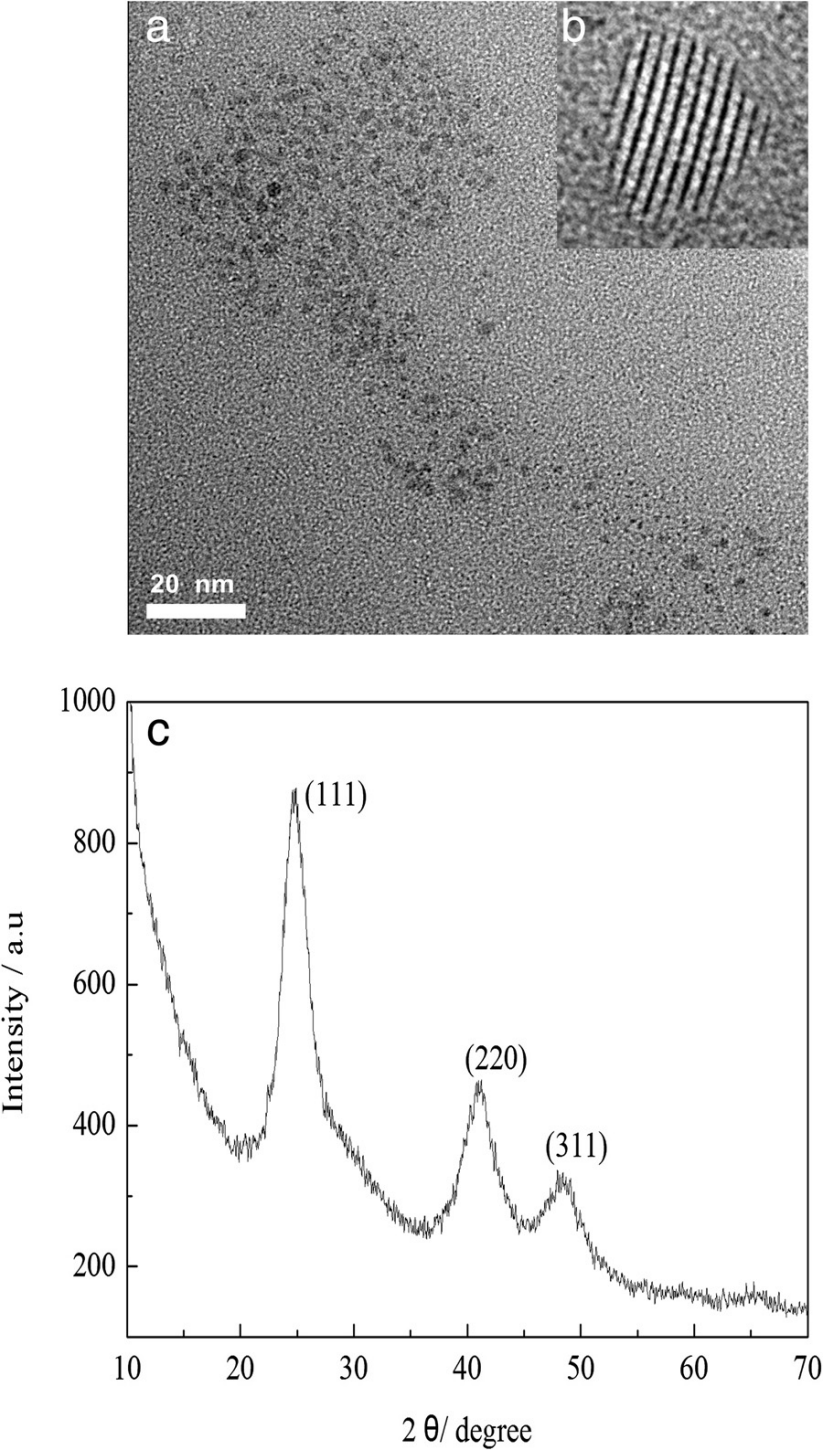
**The as-prepared CdTe QDs.** TEM (**a**) and HR-TEM (**b**) images, and XRD (**c**) pattern.

The as-prepared CdTe QDs were further characterized with XRD, TEM, HR-TEM, and XPS. As shown in Figure 3a, the diameter of the as-prepared CdTe QDs (refluxed for 120 min) is about 3 nm, which is very close to that estimated from Yu and colleagues' empirical equation [21]. Typical HR-TEM image in Figure 3b indicated good crystalline structure of the CdTe QDs. The XRD pattern of CdTe QDs (Figure 3c) shows three diffraction peaks at 24.5°, 40.6°, and 48°, which can be readily assigned to the (111), (220), and (311) planes. Such characteristic diffraction pattern is the sign of the typical zinc-blend structure (JCPDS No. 65–1046).

**Figure 4:**
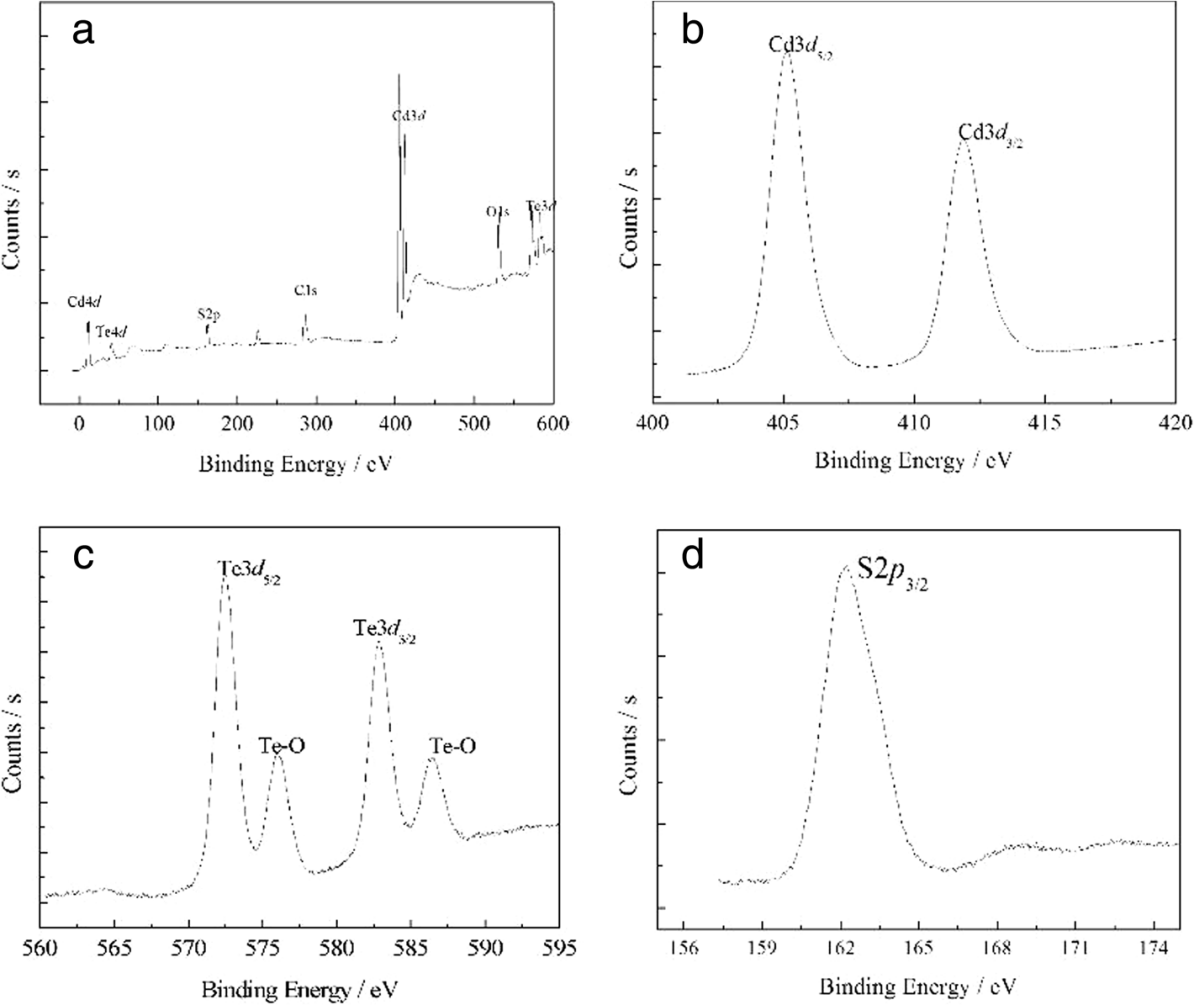
**XPS spectra of CdTe QDs.** (**a**) survey spectrum, (**b**) Cd 3*d*, (**c**) Te 3*d*, and (**d**) S 2*p.*

Figure 4 shows the corresponding elemental composition by recording XPS core level spectra. Figure 4a shows an overview spectrum of the CdTe QDs. Different Cd and Te core levels can be seen. Furthermore, the main source of carbon, oxygen, and sulfur elements was from the stabilizer MPA. In our study, we focused on the Cd 3*d*, Te 3*d*, and S 2*p* levels. The Cd 4*d* and Te 4*d* levels have not been studied here because they are quite close to the valence band and, therefore, less reliable to analyze. The spectra of the Cd 3*d* and Te 3*d* level have been recorded in Figure 4b,c. The appearances of Cd 3*d*_3/2_ peak at 411.9 eV, Cd 3*d*_5/2_ peak at 405.2 eV, Te 3*d*_5/2_ peak at 572.5 eV, and Te 3*d*_3/2_ peak at 582.8 eV confirm the existence of cadmium and tellurium species in the CdTe QDs. This is in agreement with the previous reports [22] and further confirms the formation of CdTe QDs. Moreover, it can be seen clearly in the figure that two additional peaks appeared at binding energies of 576.0 and 586.6 eV, corresponding to the Te-O bonding states in CdTeO_3_, which are possible products from the oxidation reactions of CdTe QDs [23]. As mentioned in the experimental section, the CdTe QDs are capped with MPA. So, the study of the S 2*p* level is in principle of interest, especially because some sulfur atoms originating from the organic stabilizer may have been incorporated into the CdTe lattice during the synthesis [24,25]. Figure 4d shows the S 2*p* spectrum of the CdTe QDs. The S 2*p* core level spectrum shows a single signal, where the S 2*p*_3/2_ peak appears at 162.3 eV; this may suggest that there was no sulfur incorporated into the CdTe lattice because the S 2*p*_3/2_ level in CdS has a binding energy of 161.7eV [26].

Selenite (SeO_3_^2−^) has long been known to react with thiols [27,28], we suggest that the tellurium precursor reacts in a similar manner to the selenium analogue. In this work, we explored TeO_2_ as the Te source and MPA as both the reductant for TeO_2_ and capping ligand for CdTe QDs. It has been reported that tellurite could be reduced to H_2_Te by glutathione via the GS-Te-SG complex [29]. We proposed that TeO_2_ could also be reduced to Te^2−^ in the presence of MPA as follows:

(1) $${\mathrm{Cd}}^{2+}+\mathrm{RSH}\to \mathrm{Cd}{\left(\mathrm{RS}\right)}^{+}+H^{+}$$

(2) $${\mathrm{TeO}}_{2}+2\;{\mathrm{OH}}^{-}\to {\mathrm{TeO}}_{3}{}^{2-}+H_{2}O$$

(3) $${\mathrm{TeO}}_{3}{}^{2-}+4\;\mathrm{RSH}\to \mathrm{RS}-\mathrm{Te}-\mathrm{SR}+\mathrm{RSSR}+H_{2}O+{2\;\mathrm{OH}}^{-}$$

(4) RS−Te−SR+RSH→RS−TeH+RSSR

(5) $$\mathrm{RS}-\mathrm{TeH}+\mathrm{RSH}\to \mathrm{RSSR}+{\mathrm{HTe}}^{-}+H^{+}$$

(6) $$\mathrm{Cd}{\left(\mathrm{RS}\right)}^{+}+{\mathrm{HTe}}^{-}+{\mathrm{OH}}^{-}+H^{+}\to \mathrm{CdTe}\left(\mathrm{RSH}\right)+H_{2}O.$$

In strong alkali solutions, TeO_2_ was firstly dissolved and formed TeO_3_^2-^ anion. Meanwhile, Cd^2+^ is complexed by RSH (MPA) and forms Cd(RS)^+^. In the presence of excess MPA, tellurite is first slowly formed to RS-Te-SR (3), and then the RS-Te-SR is further reduced by MPA into RS-TeH/RS-Te^−^ (4) and H_2_Te/HTe^−^/Te^2−^ (5). The CdTe QDs were obtained by the reaction between HTe^−^ and Cd^2+^ in the presence of MPA, according to reaction (6).

**Figure 5:**
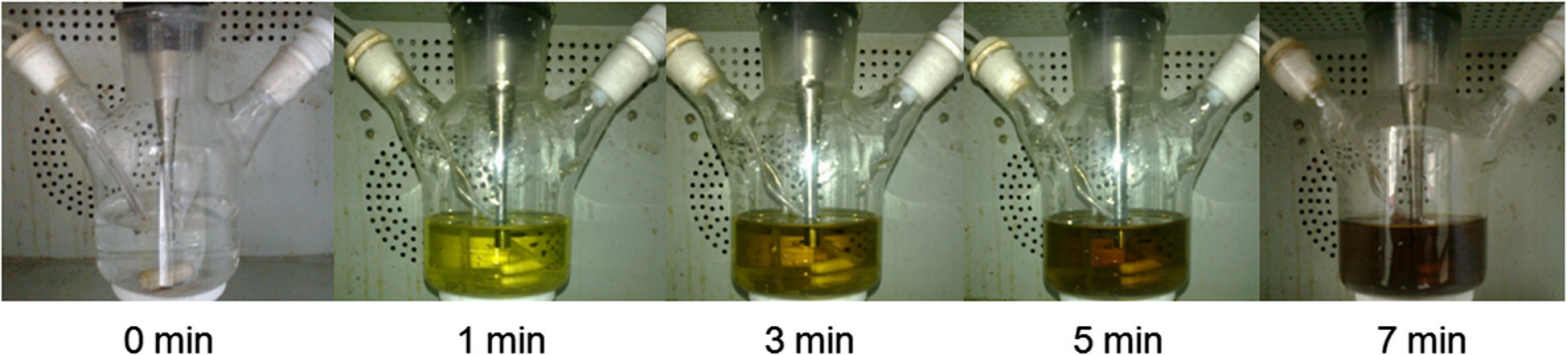
Photos of the tellurite solution after the injection of MPA.

The generation of Te^2−^ was further verified via a control experiment. As shown in Figure 5, in the absence of MPA, tellurite solution is colorless and transparent. Soon after the injection of MPA, the solution color changed to pale yellow immediately, an indication of the formation of HTe^−^. In open air condition, the solution color further changed to brown and black in about 7 min. In addition, lots of black Te precipitation was observed in the bottom of the solution due to the oxidation of Te^2−^ in open air.

**Figure 6:**
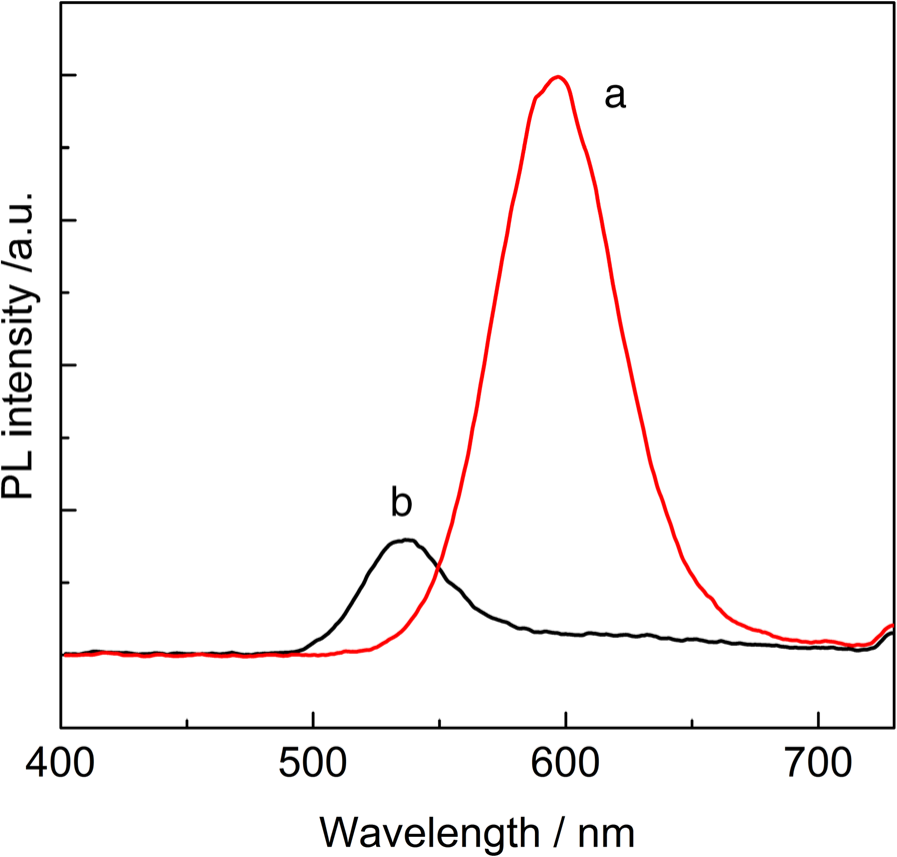
**PL spectra of CdTe QDs recorded after reaction 120 min with different reductants.** (**a**) pH = 10.0, nCd^2+^/nTe^2−^/nMPA = 1:0.25:2.7 and (**b**) pH = 10.0, nCd^2+^/nTe^2−^/nMPA/nNaBH_4_ = 1:0.25:2.7:2.7.

We further compared the use of MPA and NaBH_4_ as reductant for synthesis of CdTe QDs. As shown in Figure 6, using MPA as reductant for TeO_3_^2−^ resulted in CdTe QDs with stronger fluorescence intensity and longer emission wavelength, in comparison with those synthesized with NaBH_4_ as the reductant. NaBH_4_ is a more powerful reductant than MPA for TeO_3_^2−^. Accordingly, much more Te^2−^ ions could be generated, and more CdTe nuclei for subsequent growth of QDs. At a higher precursor concentration, more nuclei were formed, and these nuclei quickly expanded the remaining monomers with the growth of nuclei. Thus, the few remaining Cd monomers probably caused the ineffective passivation of nanocrystal surface defects, which induced the weak luminescence. However, at a lower precursor concentration, fewer nuclei were formed and relatively more cadmium monomers remained in the solution, which were favorable for surface ordering and reconstructions of CdTe QDs during the growth of QDs.

## Conclusions

In summary, a facile synthetic route for the preparation of water-soluble CdTe QDs has been proposed using 3-mercaptopropionic acid reduction of TeO_2_ directly. Since the raw materials are cheap and easy to be obtained, the synthesis process is simple, fast, and mild. The as-synthesized CdTe QDs were highly luminescent, which ensures its promising future applications as biological labels.

## Competing interests

The authors declare that they have no competing interests.

## Authors’ contributions

MS carried out the total experiment and wrote the manuscript. WJ participated in the data analysis. YH, YJ, and DH supervised the project. FL, ST, and JL provided the facilities and discussions related to them. YJ participated in the detection of the XPS and TEM. All authors read and approved the final manuscript.
